# Bilateral idiopathic orbital pseudotumour in a child: a case report

**DOI:** 10.1186/s12886-020-01718-0

**Published:** 2020-11-16

**Authors:** Fangyuan Chen, Junjie Tang, Qing Zhou

**Affiliations:** The Department of Ophthalmology, The First Affiliated Hospital of Jinan University, No. 613, Huangpu Avenue, Tianhe District of Guangzhou, Guangzhou, Guangdong Province China

**Keywords:** Bilateral, Idiopathic orbital pseudotumor, Paediatric, Case report

## Abstract

**Background:**

Idiopathic orbital pseudotumour is rare in children. We report a case of bilateral paediatric idiopathic orbital pseudotumour and review the characteristics of this case.

**Case presentation:**

A 14-year-old female patient presented at our Department of Pulmonary and Critical Care Medicine (PCCM) with complaints of recurrent severe cold and cough for 3 weeks, which had been treated with an intravenous antibiotic. Meanwhile, the patient developed swelling of both eyelids during the period of cold and cough, but her symptoms did not improve after the ocular administration of tobramycin dexamethasone eye drops. The patient was referred from the respiratory medicine ward to our department because of gradually worsening ocular pain, visual deterioration, increased intraocular pressure and serious nausea/vomiting within 24 h of hospitalization. The diagnosis of bilateral idiopathic orbital pseudotumour was made ultimately because of the course of the disease, exclusion of diagnoses such as bacterial ocular infection or malignant tumours and subsequent evidence from orbital magnetic resonance imaging (MRI). Favourable progress in the ocular tension and eyelid swelling was achieved through treatment with intravenous dexamethasone. The binocular intraocular pressure gradually declined to approximately 15 mmHg, and there was favourable progression in the patient’s vision to 20/40 in both eyes on the ninth day of hospitalization.

**Conclusions:**

Our patient developed rapidly progressive acute orbital signs and symptoms and anterior inflammation, such as pain, proptosis, limited ductions, periorbital oedema, chemosis, vision loss and high intraocular pressure. This case highlights that idiopathic orbital pseudotumour is an uncommon but important cause of acute orbital syndrome in children.

## Background

Idiopathic orbital pseudotumour (IOPT) was first described in 1905 by Birch-Hirschfeld who termed the condition orbital pseudotumor [1]. This term was used to describe orbital disease with an idiopathic cause, spontaneous resolution, and non-granulomatous changes on histopathology. While IOPT is well described among adults, it has a very low incidence in the paediatric population [1]. Therefore, little is known about the clinical presentation, and early diagnosis is a challenge.

Herein, we present a case of bilateral idiopathic orbital pseudotumour in a paediatric patient, referred from the Department of Pulmonary and Critical Care Medicine (PCCM) to our ward after a period of respiratory infection, who was diagnosed with the help of MRI and clinical characteristics, and successfully treated with intravenous corticosteroids.

## Case presentation

A 14-year-old female patient presented with photophobia, eyelid swelling, redness, and blurry vision in both eyes, along with a 3-week history of a flulike illness. Before admission to the PCCM, the patient had visited local hospitals for recurrent respiratory symptoms and eyelid swelling. The blood examination exhibited a low inflammatory response value, with a white blood cell count of 11.41*10^9/L (normal score:3.5*10^9/L -9.5*10^9/L); Erythrocyte sedimentation rate (ESR) of 34 mm/hr. (normal score: <35 mm/hr); procalcitonin (PCT) level of 0.4 ng/ml (normal score <0.1 ng/ml); and C-reactive protein (CRP) level of 10.9 mg/L (normal score:<8 mg/L) and no abnormal findings were obtained in other tests. However, treatment with antibiotics led to no remarkable improvement in the above symptoms.

**Fig. 1:**
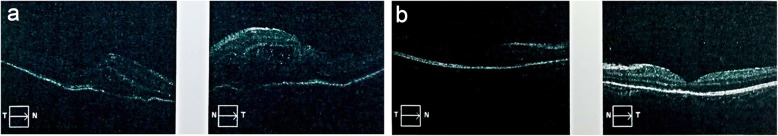
Optical coherence tomography computed tomography of both eyes. **a.** OCT showed exudative retinal detachment involving the macular area in both eyes. **b.** After treatment, OCT revealed normal peripapillary retinal nerve fiber layer thickness in left eye and no macular edema, and the subretinal fluid of right eye decreased compared to the image acquired at the first visit

On admission, the patient had dramatically decreased bilateral visual acuity, aggravated periorbital pain, and bilateral eyelid swelling accompanied by nausea, vomiting. After exclusion of neurological diseases, the patient was first examined by the ophthalmologic department of our hospital. Her right and left visual acuity were 20/100 and 20/80, respectively. The intraocular pressures (IOP) were 16 mmHg and 17 mmHg in the right and left eyes, respectively, as measured by non-contact tonometer, A slit-lamp examination revealed oedema of the eyelid (Grade 1+), and chemosis (Grade 2+) without injection. Pupils were equal, round, and reactive. Aqueous cells (Grade 1+) were present in the anterior chamber. We noticed vitreous opacity and retinal detachment in both eyes by indirect ophthalmoscopy. Optical coherence tomography-computed tomography (OCT) showed exudative retinal detachment involving the macular area in both eyes (Fig. 1, a). The visual field test showed no significant abnormality. Fundus fluorescein angiography (FFA) was not performed due to severe systemic symptoms. The clinical diagnosis of bilateral uveitis was established, and the patient was advised to receive prednisolone eye-drops (4 times daily) and cycloplegic eye-drops (twice daily).

Two days after initial presentation, the patient suffered more severer periorbital pain. Her right and left eyes had a visual acuity of 20/333 and 20/200, respectively. Her IOPs were 31 mmHg bilaterally. A slit-lamp examination showed prominent oedema of the eyelid (Grade 2+), and chemosis (Grade 2+). Mild mydriasis of both pupils, sluggish direct light reflexes, and dispersion of pigment on the anterior surface of the lens was present. Shallowness of the anterior chamber could be demonstrated and showed aqueous cells (Grade 3+) with flares. Vitreous opacity and fundus exams were not clear. This time, bilateral uveitis and secondary glaucoma (uveitis glaucoma) were diagnosed. The patient was immediately given 250 ml of 20% mannitol intravenously every 12 h and the instillation of a topical glaucoma medication (brinzolamide 1%, twice daily) to reduce the accompanying oedema in the eye.

**Fig. 2:**
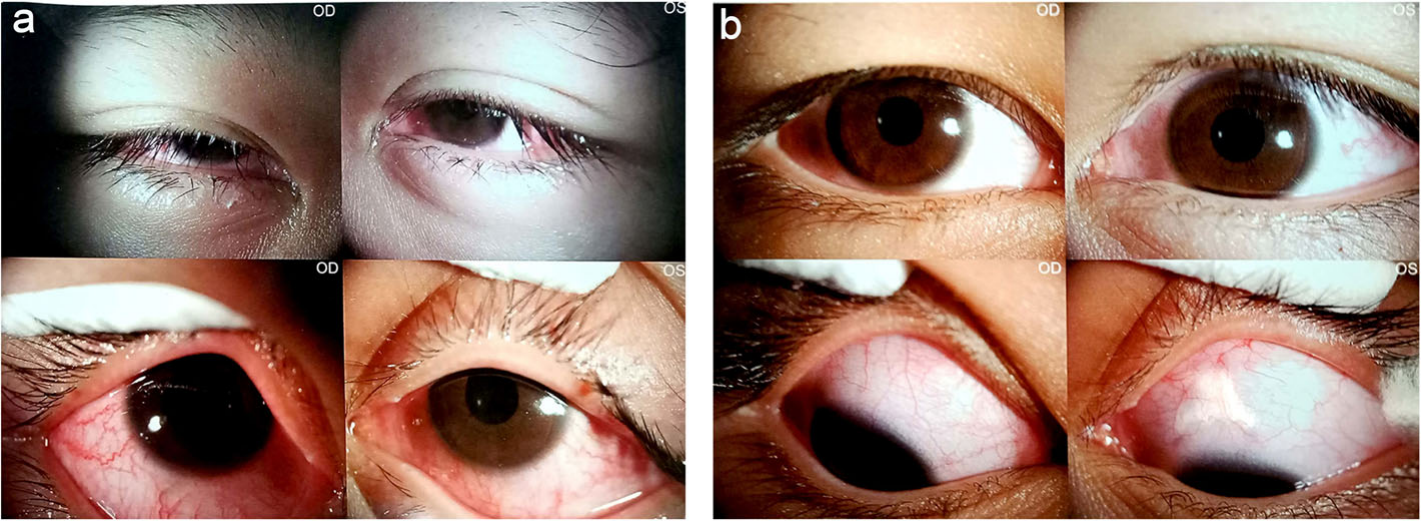
Anterior photograph of both eyes. **a*.*** *anterior* photograph taken before treatment with corticosteroids. **b.** Decreased of anterior and posterior inflammation of the anterior and posterior compared to that in the image acquired at the first visit was observed on the ninth day of hospitalization

However, several hours after treatment with mannitol, the child experienced progressive periorbital pain, gradually worsening ocular pain and visual deterioration again in both eyes accompanied by occasional serious headache along with nausea, and vomiting 3 days after admission. Examination of both eyes showed a visual acuity of finger counting at 50 cm OD, and at 20 cm OS, and the IOP had reached levels of up to 33 mmHg in the right eye and 44 mmHg in the left eye. Marked eyelid swelling with a narrowed palpebral fissure and exophthalmos was evident at this time compared to the first day of hospitalization (Fig. 2, a). Examination of the eyes revealed periorbital tenderness to palpation, and oedema of the eyelid (Grade 3+). EOMs showed slight restriction in all gazes, with painful movement. Slit-lamp examination showed diffuse epithelial oedema of the cornea in both eyes with several white keratic precipitates, inflammatory cells (Grade 3+) in the anterior chamber of both eyes, mild mydriasis of both pupils and a sluggish direct light reflex. The other tissue was not clear.

**Fig. 3:**
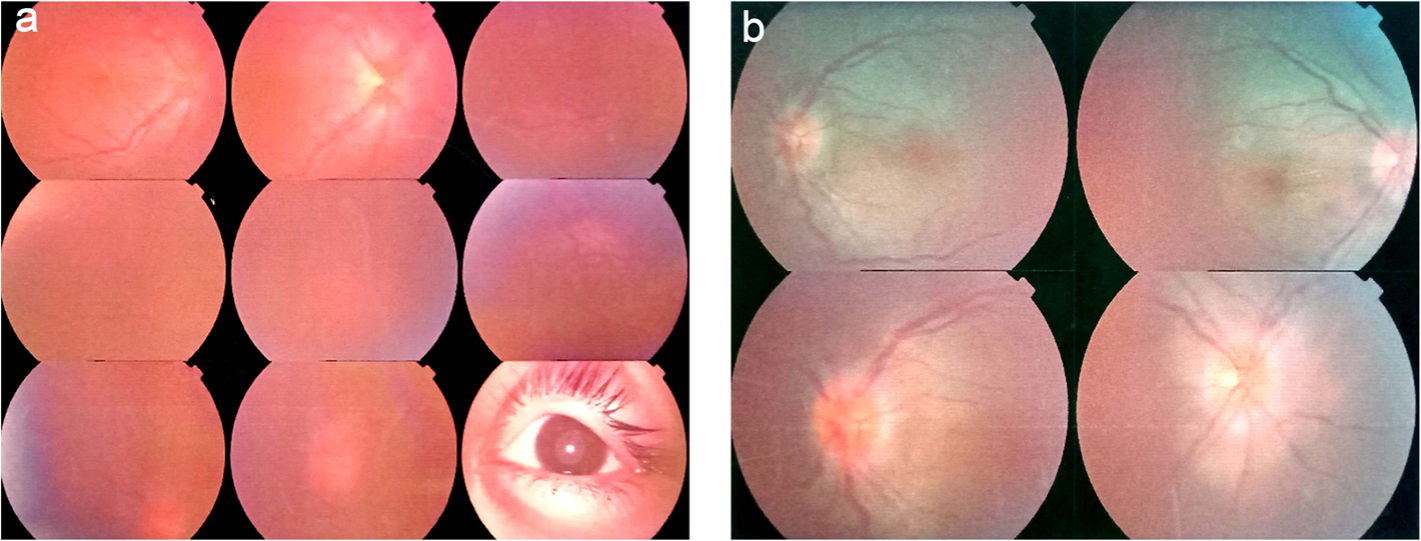
Fundus photograph of both eyes. **a.** Prior to treatment, fundus examination of the right eye showed an optic disc with blurred margins and disc oedema; similar findings were not present in the left eye. **b.** After 9 consecutive days of treatment the papilledema of both eyes was improved than before, and the macula condition recovered to normal

**Fig. 4:**
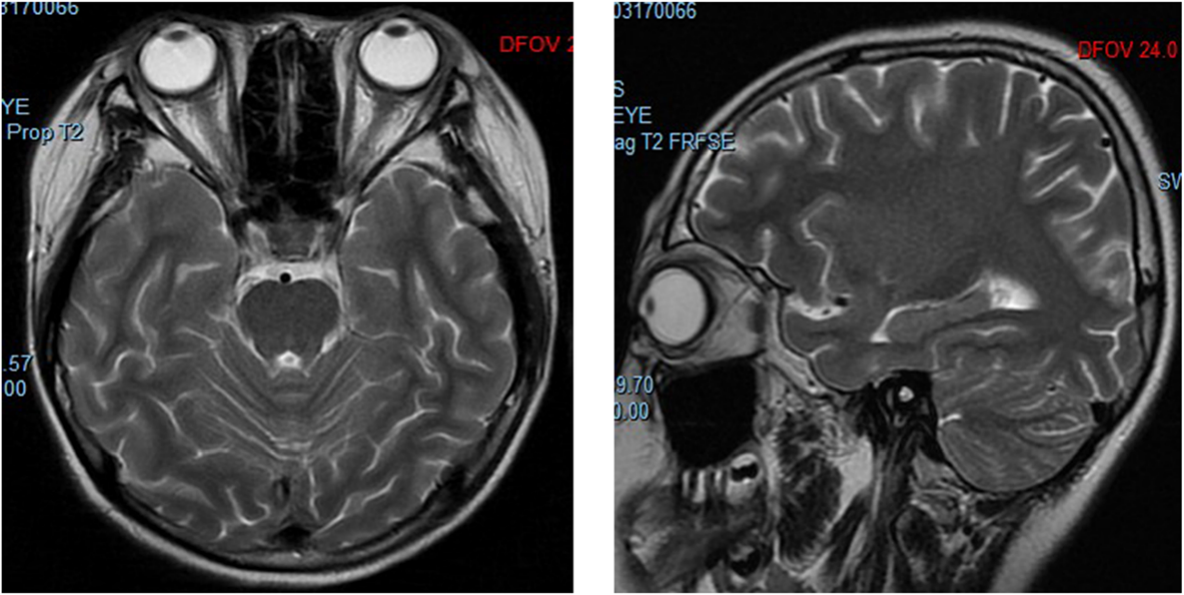
Magnetic resonance imaging. Orbital magnetic resonance imaging showed myositis, slight signal enhancement of both extraocular muscles on axial T2-weighted magnetic resonance imaging without significant change for optic nerve; soft tissue swelling and retinal detachment of the left eye. No other significant abnormality was seen in MRI of brain

**Fig. 5:**
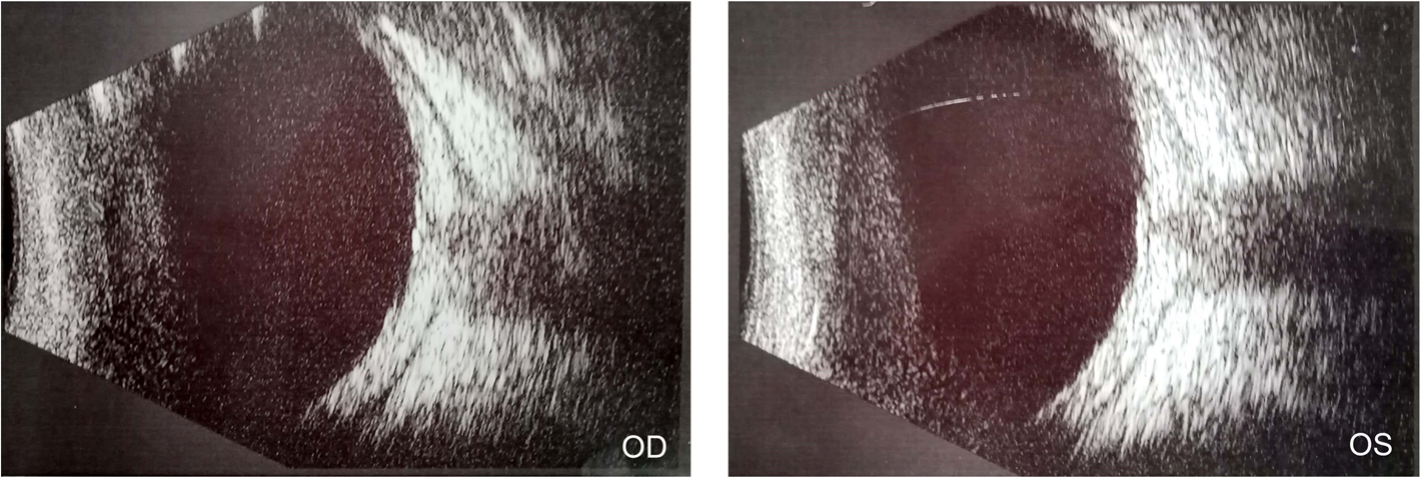
B-scan ultrasonography photograph of both eyes. During the period of treatment, exhibited vitreous haze, thickening of the eyeball wall and the “T” sign around the optic nerve wall were assessed

The abovementioned medical history, ophthalmic examinations, and auxiliary tests ruled out other possibilities such as bacterial ocular infection or malignant tumours and led to the diagnosis of bilateral idiopathic orbital pseudotumour, which was supported by evidence from orbital magnetic resonance imaging. In view of the significant decrease in visual acuity in both eyes, the patient was transferred to the ophthalmological ward and underwent intravenous dexamethasone 10 mg per day for nine consecutive days. A brisk clinical improvement was noted within 48 h of initiating steroid therapy with the child denying pain or tenderness and presenting, improved visual acuity. The right and left eyes had a visual acuity of 20/333 and 20/200, respectively, and normal IOP limits of 14 mmHg bilaterally on the second day after intravenous dexamethasone. Fundus examination of the right eye showed optic disc with blurred margins and disc oedema; similar findings were not present in the left eye (Fig. 3, a). Orbital magnetic resonance imaging (MRI) showed myositis, slight signal enhancement of both extraocular muscles on axial T2-weighted magnetic resonance imaging; and retinal detachment of the left eye. No other significant abnormality was seen in brain MRI (Fig. 4). Vitreous haze, thickening of the eyeball wall and the “T” sign around the optic nerve wall were assessed by B-scan ultrasonography (Fig. 5). The patient achieved a good outcome in ocular symptoms after approximately 1 week of intravenous dexamethasone therapy. The visual acuity improved to 0.5 bilaterally, and there was a reduction in IOP to 15 mmHg bilaterally. Decreased anterior and posterior inflammation compared to that in the image acquired at the first visit was observed on the ninth day of hospitalization (Fig. 1, b; Fig. 2, b; Fig. 3, b). The results of serologic tests (including ESR, CRP, white blood cell count, etc.) and chest X-rays were unremarkable on the ninth day of hospitalization.

The patient was discharged after 9 days of treatment with a visual acuity of 20/40 in both eyes. The left and right IOP gradually declined to approximately 15 mmHg. The patient was put on tapered prednisone and was kept under observation with monthly follow-up to rule out local recurrence or disease progression.

## Discussion and conclusions

### Aetiology

The aetiology of idiopathic orbital pseudotumour remains unknown, although several theories have been described including autoimmune disorders and upper respiratory infections. Autoimmune disorders are recognized by most scholars [1], though the antigen has not been isolated, and the good therapeutic effect of adrenocorticosteroids on this disease may support this theory. Idiopathic orbital pseudotumour have been associated with several infections and flulike viral illnesses, but the exact nature of these associations is not clear; in this case, the patient had an obvious history of upper respiratory infections for the past 3 weeks. Genetic and environmental factors have also been suggested [2].

### Incidence

Orbital pseudotumour is the third most common ophthalmologic disease of the orbit, accounting for 8–11% of all orbital tumours. IOPT has historically shown no sex or racial predilection but is rare in childhood [3, 4]. However, most cases reported in the literature appear to be diagnosed in adults, typically middle-aged individuals; paediatric cases account for only 6–17% of cases [5],among which bilateral cases account for one-third of the cases in the paediatric population [6]. Until now, the youngest patient with idiopathic orbital pseudotumour has been reported in the literature to be 3 years of age [7].

### Clinical presentation

Idiopathic orbital pseudotumour (IOPT) can involve any of the orbital soft tissues with the rectus muscles (myositis) and the lacrimal gland (dacryoadenitis) being the most commonly involved sites. Clinically, IOPT is often classified into two types based on the different anatomical sites of orbital involvement and the time of onset. Type 1is characterized as: diffuse: including acute and chronic types, such as acute diffuse orbital inflammatory pseudotumour; type 2 is characterized as focal: including myositis, dacryoadenitis, periscleral inflammation and peripapillary neuritis. Acute diffuse acute orbital inflammatory pseudotumour ware present in this case. Our patient experienced an initially acute onset and was found to have marked eyelid swelling with narrowed palpebral fissure, periorbital tenderness, periorbital oedema, proptosis, chemosis, iritis and “T” sign, which were disclosed in B-scan ultrasonography. These findings relate to the classification of diffuse acute orbital inflammatory pseudotumour.

Reportedly, IOPT typically presents with acute orbital signs and symptoms in the pediatric population, such as sudden or slow and progressive onset of pain, eyelid oedema, proptosis and subconjunctival haemorrhage [8]. Meanwhile, vision loss, photophobia, limited ductions with diplopia and system symptoms such as, headache, vomiting and weight loss are well described among children, it has a very low incidence in adults [6, 8]. It is also reported to differ from IOPT in adults in that affected children more often have bilateral involvement, iritis and a history of trauma preceding inflammation [4, 9].

In the acute phase of inflammation, previous studies have demonstrated that choroidal effusion followed by forward displacement of the irislens diaphragm, and oppression of the eyeball may increase intraocular pressure. In this case, the patient experienced worsened bilateral orbital pain after treatment with intravenous mannitol, and had the clinical manifestation of secondary glaucoma. The symptoms were not relieved, but the ocular pain was aggravated. We hypothesized that the decreased pressure gradient between the lesion-orbital tissues after treatment with the dehydrating agent may have led to aggravation of acute oedema; or the breakdown of the blood-retinal barrier and increased vascular permeability, causing mannitol to accumulate in soft tissue and ultimately resulting in elevation of the intraocular pressure and exacerbation of ocular oedema because of reverse osmosis. However, cases of the severe side effects of mannitol have not been reported and the underlying mechanisms require further study.

### Diagnosis and differential diagnosis

Idiopathic orbital pseudotumour is a diagnosis of exclusion and can present with a variety of symptoms; therefore, it is paramount to approach the evaluation with a thorough differential diagnosis. Especially in the acute phase, IOPT can present the ocular symptoms similar to Vogt-Koyanagi-Harada disease (VKH), like retinitis, macular oedema, optic disc oedema and serous retinal detachment. That is also the reason why IOPT is easily misdiagnosis. However, VKH generally does not involve the extraocular soft tissues and ocular muscles, and the eye movements are not deficit. Pain with eye movements, periorbital edema, palpable mass, and increased intraocular pressure are not common in VKH. Therefore, imaging studies, computed tomography (CT) and magnetic resonance imaging (MRI) help identify underlying disease [10], however, serological tests may be required to exclude a systemic cause. In the paediatric population, malignancies such as rhabdomyosarcoma, retinoblastoma, chloroma, and neuroblastoma should be considered in the differential diagnosis of patients with bilateral disease [11].

Biopsy is not mandatory in all cases and should be reserved for patients with refractory or rebound inflammation after treatment with corticosteroids, patients with a poor response to corticosteroids or patients for whom there is concern regarding an abscess or malignancy [11, 12]. It is suggested that systemic causes should be ruled out and a biopsy should be performed. In this case, the parent of child refused the biopsy when other eye hospital suggested, and the patient’s symptoms were significantly improved after 1 week of corticosteroid therapy, thereby avoiding the risks posed by biopsy, such as reductions in visual acuity, persistent proptosis, extraocular muscle paresis, and restrictions [6, 13].

### Treatment

Corticosteroids are the mainstay of treatment and the effective rate is 30–80% [14];however, high incidence of recurrence has been reported to occur in up to 33–58% of patients following resolution of their initial event [8, 15–18]. Rapid response to steroids is also considered diagnostic. Prednisone was given the tapering dose began at 1–1.5 mg/kg/day in children, which lead to a brisk clinical resolution within 24–48 h. In our case, we obtained an excellent response with steroids compared to intravenous antibiotics. However, adverse effects may limit the long-term use of this modality in the paediatric population.

Alternative treatments, including low-dose (10–30 Gy) radiotherapy delivered over 2–3 weeks and immunosuppressive chemotherapy may be beneficial in population with corticosteroids fail or medically contraindicated, or recurrences when the patient is already receiving corticosteroid therapy [12].

## Conclusions

Idiopathic orbital pseudotumour in childhood can have a low incident and diverse presentation, so it is often misdiagnosed as orbital cellulitis or as an orbital mass with conjunctivitis. It is imperative to diagnose and treat these patients in a timely and effective manner, in ease of visual improvement. In this case, the patient had a favourable outcome with improved visual acuity and was discharged on an oral steroid taper and prednisolone drops. She was then followed up for another six months to observe any recurrence.

## Data Availability

All data have been presented within the manuscript and in the form of images.

## Abbreviations

IOPT: Idiopathic orbital pseudotumour
PCCM: Department of pulmonary and critical care medicine
IOP: Intraocular pressure
FFA: Fundus fluorescein angiography
OCT: Optical coherence tomography-computed
VKH: Vogt-koyanagi-harada disease
CT: Computed tomography
MRI: Magnetic resonance imaging
